# The Absence of Permanent Sensorineural Hearing Loss in a Cohort of Children with SARS-CoV-2 Infection and the Importance of Performing the Audiological “Work-Up”

**DOI:** 10.3390/children9111681

**Published:** 2022-11-01

**Authors:** Rita Malesci, Davide Rizzo, Valeria Del Vecchio, Nicola Serra, Giuseppe Tarallo, Domenico D’Errico, Valentina Coronella, Francesco Bussu, Andrea Lo Vecchio, Gennaro Auletta, Annamaria Franzè, Anna Rita Fetoni

**Affiliations:** 1 Section of Audiology, Department of Neuroscience, Reproductive Sciences and Dentistry, University of Naples Federico II, Via Pansini, 5, 80131 Naples, Italy; 2 Otolaryngology Division, Department of Medical, Surgical and Experimental Sciences, University of Sassari, Viale San Pietro, 43, 07100 Sassari, Italy; 3 Department of Public Health, University of Naples Federico II, Via Pansini, 5, 80138 Naples, Italy; 4 Section of Pediatrics, Department of Translational Medical Sciences, University of Naples Federico II, Via Pansini, 5, 80138 Naples, Italy; 5 Department of Otolaryngology Head and Neck Surgery, Università Cattolica del Sacro Cuore, Largo A. Gemelli, 8, 00168 Rome, Italy

**Keywords:** COVID-19, SARS-CoV-2, genetic hearing loss, permanent hearing impairment, audiological surveillance, children

## Abstract

Background: Currently, the novel coronavirus (SARS-CoV-2) causes an acute respiratory illness named COVID-19 and is a controversial risk factor for hearing loss (HL). Herein, we aim to describe the associated symptoms and to evaluate hearing function in the COVID-19 pediatric population. Methods: A retrospective cross-sectional observational study was carried out on 37 children who contracted COVID-19 infection with no previous audio-vestibular disorders. Clinical data on the infections were collected, and an audiological assessment of all affected children was performed by using different diagnostic protocols according to their age. Results: Fever, upper respiratory and gastrointestinal manifestations were common presentations of infection. Audiological function was normal in 30 (81.08%) children, while 7 children showed an increased hearing threshold: 6 (16.21%) had transient conductive hearing loss (CHL) due to middle ear effusion and normalized at the follow-up and 1 had sensorineural hearing loss (SNHL). A single child was affected by bilateral SNHL (2.7%); however, he underwent a complete audiological work-up leading to a diagnosis of genetic HL due to a MYO6 gene mutation which is causative of progressive or late onset SNHL. Conclusions: HL needs to be considered among the manifestations of COVID-19 in children, nevertheless, we found cases of transient CHL. The onset of HL during or following COVID-19 infection does not eliminate the indication for maintaining audiological surveillance and audiological work-ups, including genetic diagnosis, to avoid the risk of mistaking other causes of HL.

## 1. Introduction

The outbreak of a novel Coronavirus, called Severe Acute Respiratory Syndrome Coronavirus 2 (SARS-CoV-2) is currently a major global health concern [1,2]. Although there is limited data in children, emerging evidence suggests a greater risk in children than initially predicted [3]. At the present time, it is also controversial whether the spreading of SARS-CoV-2 may be considered a risk factor for Permanent Hearing Impairment (PHI) given that infections are common reasons for congenital or acquired hearing loss (HL) [4]. Therefore, the current literature aims to clarify the relationship between HL and SARS-CoV-2.

Several systematic reviews focused on audio-vestibular disorders in adults [5,6,7] and reported these symptoms in 7–15% of patients with Coronavirus Disease 19 (COVID-19) [6]. The most common symptom is tinnitus, followed by hearing impairment including conductive, sensorineural, and mixed and rotatory vertigo [6]. HL was associated with different clinical presentations, including a case of a patient with severe illness related to pneumonia and mental dysfunction who had received ototoxic medicaments [8] and asymptomatic subjects without other causes such as metabolic diseases, ototoxic drug usage, history of trauma, and history of upper respiratory tract infections [9].

Indeed, the pathophysiology of audio-vestibular disorders related to COVID-19 is still unknown [6], although potential mechanisms have been proposed as cochleitis or neuritis [10], cross-reactions [10], vascular disorders [11,12], immune-mediated disorders (e.g., overzealous production of pro-inflammatory cytokines) [8]. Currently, it is suggested that SARS-CoV-2 is a neurotropic virus [13,14] that can extend to the cranial nervous system by anterograde and retrograde transport by way of motor proteins (kinesins and dyneins) through sensory and motor nerve endings [15]. Furthermore, ACE-2 receptors may be targeted by SARS-CoV-2 because they are expressed in glial tissues, neurons, and brain vasculature [16,17].

Nevertheless, studies on a complete evaluation of audio-vestibular function in COVID-19 patients compared with control groups are lacking [6]. In addition, the aim of increasing the follow-up and improving etiological diagnosis in order to exclude other possible causes of audio-vestibular symptoms is challenging.

The literature described mainly cases in adult age groups, with very few articles concerning the pediatric population [3,18,19]. Children seem to be less susceptible to this infection [20], they are usually asymptomatic or manifest mild clinical symptoms compared to adults [19,21], and have a good prognosis [20,22]. However, the literature suggests there is a risk of multisystem inflammatory syndrome in children (MIS-C), a rare post-infectious hyper-inflammatory disorder associated with SARS-CoV-2 [23].

To the best of our knowledge, few studies [15,24,25,26,27] have focused on hearing function in children. Namely, in a study performed on COVID-19-infected subjects, including newborns exposed to intrauterine SARS-CoV-2 and children from the perinatal period to adolescence, no HL or central auditory processing disorders was found [28]. In a recent study, newborn hearing screening (NHS) outcomes of neonates born to women who were SARS-CoV-2 positive during pregnancy were compared with neonates born to healthy women [15]. This study suggested that COVID-19 infection increases the “fail” rate of NHS without affecting the overall outcomes of NHS that could be related to the growth restriction, preterm birth, and perinatal mortality observed in these neonates [15].

Thus, these infections represented a risk factor for NHS failure as a consequence of maternal infection, leading to fetal distress more than the infection per se, although COVID-19 could not be excluded as a risk factor for hearing loss [15].

However, this study was conducted in a small sample size and further studies on children are desirable. Therefore, the aim of this work is to describe the clinical presentation, audiological assessment, and outcomes in a sample of children affected by COVID-19. Furthermore, as shown in our case series, the COVID-19 pandemic requires maintaining audiological surveillance in suspected clinical cases without missing that other etiologies of PHI can co-exist and that it must be considered in the audiological work-up, including genetic diagnosis.

## 2. Materials and Methods

A retrospective cross-sectional observational study was carried out on 37 children who contracted COVID-19 and who were evaluated in the Unit of Audiology of the Department of Neuroscience, Reproductive Sciences and Dentistry of the University of Naples Federico II. The study was performed from October 2021 to March 2022.

This research project was approved by the University of Naples Federico II Ethics Committee (protocol number 272/21 on 7 October 2021). All procedures involving human participants were in accordance with the ethical standards of the institutional and national research committee and with the 1964 Helsinki Declaration and its later amendments, or comparable ethical standards. The parents of all children involved in the study gave their informed consent to the use of anonymized data.

All children included in this study underwent COVID-19 diagnosis based on a positive nasopharyngeal swab using real-time polymerase chain reaction (PCR).

Criteria for inclusion in this study were the absence of previous HL, bilateral pass responses at TEOAEs (transient evoked otoacoustic emissions) at birth, no risk factor for PHI, absence of marked adenotonsillar hypertrophy and recurrence of otitis media with effusion, and other associated comorbidities.

The colleagues of the pediatric unit suggested to all parents an audiological evaluation in order to bring all of the COVID-19 children in for an assessment for the purpose of the research question. Following the criteria for inclusion, the colleagues of the pediatric unit selected patients to refer to the audiology unit in order to bring all of the COVID-19 children without other comorbidities or risk factors for PHI in for an assessment for the purpose of the research.

During the enrolment period, 50 families were approached because we excluded children suffering from other comorbidities and/or having risk factors for PHI to avoid bias. Among recruited children, 37 families decided to be enrolled and 13 declined participation.

We collected demographic data such as age, sex, and family and medical history from pediatric reports, including the symptom presentation, signs, disease severity, treatments during quarantine, and clinical course in cases of hospitalization. An audiological evaluation was performed within 1 month after testing negative for COVID-19 using real-time PCR.

Demographic data of children who underwent audiological evaluation are reported in Table 1: the ages of included children ranged between 10 months and 17 years of age.

Twenty-seven infants (13 males and 14 females) were <4 years of age (Group A) and the mean age was 19.96 ± 8.79 months. Additionally, 10 children (3 males and 7 females) were ≥4 years of age (Group B) and the mean age was 11.6 ± 3.86 years.

### 2.1. Pediatric Evaluation

All symptoms and signs of the acute stage of the disease were collected from the electronic medical record of children who underwent diagnosis and clinical support in the Pediatric Unit of this hospital and were addressed to our audiological evaluation. Therefore, all procedures including physical examination and diagnostic testing were performed by pediatricians and we described only their clinical reports.

We concentrated on the most common symptoms declared, such as fever, astenia, vomiting, diarrhea, rhinorrhea, sore throat, cough, dyspnea, rash, and hyposmia, in addition to the major complications of MIS-C, vasculitis, and the treatment carried out, if available.

According to the published criteria [21], based on clinical, laboratory and radiological profiling, symptomatic SARS-CoV-2 infections can be graded as mild, moderate, severe, or critical. Among COVID-19-related major complications, MIS-C [29] and Vasculitis [30] were described according to the literature. MIS-C is a life-threatening situation characterized by fever for at least 24 h, laboratory evidence of inflammation, evidence of clinically significant illness requiring hospitalization with multisystem involvement, evidence of prior or current SARS-CoV-2 infection, and no alternative plausible diagnosis [29].

Furthermore, we identified children who had not received medical treatment and in Table 2 we showed the types of treatments used in both groups as: non-ototoxic antibiotics, paracetamol, steroids, immunosuppressants, and heparin. From the analysis, no significant differences among the types of treatments used in both groups were observed (*p* = 0.24 for Group A and *p* = 0.081 for Group B). Children in Group B more frequently received non-ototoxic antibiotics and heparin than younger children.

### 2.2. Audiological Evaluation

All families who approved to have a diagnostic assessment of their children were referred to the Audiology Unit. The diagnostic battery used to diagnose hearing loss is shown below.

Different audiological protocols were adopted to evaluate the children according to their age, and in all enrolled subjects the external and middle ear evaluation with otoscopy was performed. The objective instrumental evaluation was used for infants between 0 and <4 years of age and subjective instrumental evaluation was used in children between ≥4 and 17 years of age. The difference was due to the difficulty in having the same assessment in younger and older children to accurately study hearing function on both sides. Therefore, TEOAE, click-evoked auditory brainstem responses (ABR) and tympanometry registration were performed on babies in Group A, and TEOAE, behavioral audiometry or pure tone audiometry, and tympanometry were performed on children of Group B. All audiological procedures were performed by the same pediatric audiologist.

TEOAE and ABR were performed during spontaneous sleeping in a soundproof and faradized room. The device used for TEOAE was an Accuscreen^R^ Madsen (by Natus, Montegrotto Terme, Italy) newborn hearing screener, an automated tool whose output simply indicates the response score of “pass” or “fail”. The TEOAE test was performed on each side and its evaluation was based on noise-weighted averaging counting of significant signal peaks; stimuli were non-linear click sequences at 35 dB nHL with a frequency range of 1.5–4.5 kHz.

The device used for click-evoked ABR was Neuro-Audio (by Inventis, Padova, Italy). The test was performed by using a three-electrode montage with impedance kept at ≤3000 dines. The active electrode was applied to the forehead, the exploring electrode was placed on the homolateral mastoid, and one was a contralateral mass electrode.

The standard procedure consists of alternate clicks at 21 pps, duration 0.1 ms, filter settings 100–2000 Hz, and analysis time 12 ms. The protocol starts with monaural stimulation at 80 dB nHL for the identification of the three main waves I, III and V, decreasing the stimulation by 10-dB steps to a minimum of 20 dB nHL. Normal hearing was defined based on the presence and persistence of V wave for acoustics stimuli < 30 dB nHL. The diagnosis of HL was defined as the presence and persistence of V wave for acoustic stimuli ≥ 30 dB nHL.

Tympanometry was performed with Resonance^®^ R36M (Resonance^®^, Gazzaniga, Italy) with a 226 Hz probe tone. Normality criteria for tympanometry was a sharp peak with middle ear pressure in the range of −150 to +25 daPa, static compliance between 0.2 and 0.9 mL and ear canal volume with a normal range (0.4 mL to 1 mL). A reduced or no measurable middle ear pressure with normal ear canal volume suggested a middle ear dysfunction.

Various audiometric procedures (visually reinforced audiometry, conditioned play audiometry, and conventional audiometry) were adopted to identify the pure tone threshold using frequencies from 0.125 to 8 kHz with the device Resonance^®^ R37A (Resonance^®^, Gazzaniga, Italy).

According to the Bureau International for Audiophonology (Biap) classification, the severities of HL were: normal (<20 dB nHL), mild (21–40 dB nHL), moderate (41–70 dB nHL), severe (71–90 dB nHL) and profound (>91 dB nHL) [31].

### 2.3. Statistical Analysis

Continuous data were expressed as mean ± standard deviation (SD), or median with Interquartile Range (IQR), while categorical variables were expressed as numbers or percentages.

The chi-square test and Fisher’s exact test were performed to evaluate significant differences between Group A and Group B regarding qualitative variables such as gender, symptom type and hearing loss. In particular, Fisher’s exact test was used where the chi-square test was not appropriate.

Cochran’s Q test was used to evaluate significant differences among symptoms or treatments for each group. If the Cochran’s Q test was positive (*p*-value < 0.05), then the Minimum Required Differences method post hoc test with Bonferroni *p*-value corrected for multiple comparisons according to Sheskin [32] was used to evaluate the symptom or treatment which appeared significantly more frequently in each group.

Testing for normal distribution was performed by Shapiro–Wilk test. The t-test was used to compare the means between two groups, while for sample data not normally distributed the Mann–Whitney test was used, such as for the age variable.

All tests with *p*-value(*p*) < 0.05 were considered significant. The statistical analysis was performed by Matlab statistical toolbox version 2008 (MathWorks, Natick, MA, USA) for Windows 32-bit.

## 3. Results

In Table 1, the impact of disease in both groups of children is reported, showing no significant difference between Group A and B based on gender (*p* = 0.46), symptoms (*p* = 0.36), major complications (*p* = 0.29), percentage of children hospitalized (*p* = 0.46) and treatment (*p* = 0.27). The symptoms were usually mild, and it was reported in 23/27 (85.19%) babies in Group A and 7/10 (70%) children in Group B.

As shown in Table 3, the most common symptom was fever in both groups and no statistical significance was detected between younger and older children concerning symptoms.

Statistical analysis for each group showed a significantly high occurrence of children with fever (70.37%), compared to other symptoms considered such as asthenia (11.11%), vomiting (18.52%), diarrhea (18.52%), rhinorrhea (33.33%), sore throat (14.81%), dyspnea (7.41%), and rash (7.41%) in Group A. Indeed, a significant difference was detected between cough and hyposmia. However, we were not able to investigate smell disorders such as hyposmia among the children of this group due to their age and their low compliance in referring to their symptoms. Instead, in Group B, there was a high occurrence of children with fever but with no statistical significance with other symptoms declared.

Nevertheless, 13 (48.15%) from Group A and 3 (30%) from Group B were hospitalized for acute respiratory symptoms. Four patients had major complications: MIS-C in three children (one from Group A and two from Group B) and cerebral vasculitis in one baby from Group A.

Moreover, in Table 2 we showed the types of treatments used in both groups. In our analysis, no significant differences among the types of treatments used in both groups were observed (*p* = 0.24 for Group A and *p* = 0.081 for Group B). Children in Group B more frequently received non-ototoxic antibiotics and heparin than younger children.

For the audiological assessment, all participants were evaluated after quarantine and/or hospitalization, when COVID-19 was undetectable and within 1 month of COVID-19 negativity. As shown in Table 4, 30 (81.08%) children exhibited normal hearing while 7 (18.92%) children showed increased hearing levels. In six cases (16.21%), a conductive hearing loss (CHL) was found, HL was bilateral in four infants, in two cases there was unilateral HL without differences between groups, and one case of SNHL was found (1/37; 2,7%). HL was found in both groups without significant differences (*p* = 0.07).

Namely, CHL was mild in four children (three bilateral and one unilateral) and moderate in two children (one bilateral and one unilateral) as shown in Table 5.

All CHL were due to middle ear effusion. OME was diagnosed following these criteria: a reduced or non-measurable middle ear pressure with normal ear canal volume and the presence of middle ear effusion upon otomicroscopic observation. In all cases, the medical history indicated the early onset of CHL during COVID-19 infection. All children recovered at 2 months follow-up after local treatment with nasal decongestants, and aerosol therapy with a combination of corticoids and mucokinetic agents. In a boy child (Group B) 10 years of age, bilateral SNHL was diagnosed, exhibiting moderate HL for the higher frequencies as described. Remarkably, he referred to difficulties understanding words during online learning after COVID-19 infection. No tinnitus or vertigo was referred to. Pure tone audiometry showed symmetrical moderate high frequencies SNHL and the speech recognition score was 100% on both ears using phonetically balanced words at 60 dB nHL bilaterally. TEOAE and ABR measurements were negative for retro-cochlear pathology. Thus, a sudden SNHL related to COVID-19 infection could be suspected and clinical investigations were performed. Concerning his NHS, the responses of TEOAEs were bilaterally a pass. Both autoimmune and viral screens were negative. Pre- and post-gadolinium-enhanced brain magnetic resonance imaging (MRI) showed the absence of intracranial appearances and abnormalities of the internal auditory meatus or cerebellopontine angles. Comprehensive genetic testing using massively parallel sequencing or next-generation sequencing showed a heterozygous missense mutation of p.Arg980Cys (c.2938C > T) in exon 27 of the *MYO6* gene. According to the geneticist’s interpretation, this mutation could be considered of uncertain significance if not associated with phenotype or family recurrence. Thus, the family was called to deepen counseling and the mother—who previously denied the presence of HL in the family—after the proband’s genetic diagnosis, stated that she was affected by progressive hearing impairment. Thus, all family members underwent pure tone audiometry and a symmetrical moderate high frequencies SNHL was found in the mother. Therefore, genetic analysis was extended to the family. The genetic consultation confirmed the mutation of p.Arg980Cys (c.2938C > T) in exon 27 of the *MYO6* gene in the mother and the seven-year-old younger sister. Therefore, the presence of the heterozygous identified mutation is likely to be compatible with the clinical phenotype of a late onset/progressive HL reported in the patient and mother in the autosomal dominant inheritance pattern. Mother and child have been treated with hearing aids. Conventional amplification hearing aid has been used to rehabilitate HL in the proband, with good improvement in speech perception and academic performances.

## 4. Discussion 

Despite the emerging burden of the SARS-CoV-2 pandemic, as previously reported, children have a lower incidence of COVID-19 infection and our findings confirm a less severe clinical presentation as compared to adults [27,33]. Furthermore, it is still unknown whether COVID-19 has effects on hearing function in pediatric populations [6,7]. Herein, we confirm the occurrence of mild symptoms in a cohort of infants and hearing impairment according to data from previous pediatric systematic reviews [3,18]. We evaluated children from the neonatal period to adolescence and our results found that no significant differences were observed between infants <4 years of age (Group A) and those ≥4 years of age (Group B) based on gender, symptoms, major complications, and percentage of hospitalized children. Interestingly, younger children (Group A) manifested a significantly high occurrence of fever, while no significant differences in symptoms were observed in Group B. Moreover, the percentage of children hospitalized (*p* = 0.46) was higher in Group A than in Group B. An increased occurrence of hospitalization in younger children is due to greater worry in the family concerning the early age of the onset, even if the symptoms were usually mild. Furthermore, despite a higher hospital course in Group A, treatment was more frequently reported in Group B. The latter received all common medicines used in COVID-19 such as non-ototoxic antibiotics and heparin [34]. The differences between the two groups could be explained by greater caution in prescribing medicine in Group A, considering the mean age of 19.96 ± 8.79 months.

We found the presence of HL after COVID-19-negative testing in about 18% of cases, however, HL was transient. The pediatric surveillance of the enrolled children and the presence of hospitalized children may explain the increased number of patients with HL. Transient CHL was found without significant differences between age groups and related to middle ear otitis with effusion. The middle ear inflammation is indeed a complication of Eustachian tube dysfunction that occurs during a viral upper respiratory tract infection and then it could be independent of COVID-19 etiology and related to manifestations of viral infection, including fever and nasal symptoms.

Nevertheless, in our sample, we have excluded the children with marked adenotonsillar hypertrophy and recurrence of OME. On the other hand, the literature has described a reduction in OME incidence during the pandemic year as a consequence of social distancing and the adoption of facial masks [35]. Furthermore, a history of chronic otitis media can lead to auditory processing disorders [36]. Its early diagnosis, prevention of recurrence, and audiological surveillance are important for minimizing the effect on child development. In our sample, we detected transient conductive hearing loss that recovered completely after therapy, although we have recommended audiological surveillance related to this hypothesis. In our preliminary results, the percentage of OME is quite high if we consider our exclusion criteria. Major restrictions decreased the incidence of the most common viral infections and SARS-CoV-2 was often the etiology of upper airway disorders and consequences such as OME. These audiological outcomes also require surveillance in order to prevent recurrence and other related clinical conditions.

The presence of anosmia or hyposmia is considered a possible indicator of COVID-19 infections in adults [37]; however, this is difficult to detect in children due to poor compliance and the lack of standardized tests, especially in younger and non-collaborative children.

Remarkably, in our cohort, a child with bilateral SNHL was detected. A sudden SNHL related to COVID-19 could be suspected based on the apparent sudden onset, which appeared as poor language intelligibility during distance education. However, we decided to apply our protocol of diagnosis in this case, including genetic investigation, which permitted us to find a genetic etiology due to a mutation in the MYO6 gene. This gene mutation may be associated with both autosomal dominant and recessive types of non-syndromic HL, congenital and late-onset, although this involvement is uncommon and limited to a few families worldwide [38]. The MYO6 gene [39] is located on chromosome 6q13, which contains 32 exons and spans 70 kb. It encodes Myosin VI, which is an actin-based molecular motor necessary for the functioning of inner ear hair cells [38]. Recent guidelines suggest the importance of genetic testing in the evaluation of patients with PHI as part of an initial work-up if a known family history exists [40]. Although the family history might appear negative due to a reluctance to face stigma, we underline the importance of evaluating the risk of hereditary HL in childhood for the risk of progressive and late-onset even in the presence of different risk factors for HL.

In conclusion, this work is a preliminary study for exploring the role of COVID-19 on hearing function. Our experience with these preliminary results seems to not confirm COVID-19 infection as a risk factor for PHI in children. A major limitation of this study is the relatively small sample size, especially for Group B. Particularly, since the number of participants of the groups was not equal, we considered the ratio 1:3. This choice was due to limited compliance of family, mainly among Group B. Even today, parents do not always accept coming to our outpatient clinic when they do not suspect HL. They usually prefer to postpone audiological evaluations because the risk of infection is perceived as being high. Some statistical analyses were performed on small sample data, increasing the probability of statistical bias. To reduce the presence of statistical bias, we used statistical tests for small samples or continuity corrections. Therefore, our data may be considered preliminary results requiring multicentre studies in order to better understand the association between infection and both CHL and SNHL in this ongoing pandemic. According to our findings, CHL described as middle otitis is the most common ear pathology related to COVID-19 infection [41]. Despite it being a self-limited condition, misdiagnosis of mild to moderate HL during childhood can affect speech and language development and scholastic performance, indicating the value of an opportunity for a prompt diagnosis. Previous studies on hearing function in children after COVID-19 infection [28] and on infants exposed to SARS-CoV-2 intrauterine [15,24,25] suggest the absence of SNHL as a manifestation of childhood infection. Two studies reported a significant reduction in the TEOAE amplitude both in adults [42] and newborns [15], indicating that outer hair cells could be a target of the virus. Interestingly, a study performed on human and mouse inner ear cells reported that they have the molecular machinery to allow SARS-CoV-2 entry leading to cochlear and vestibular dysfunction [17]. Nowadays, an accurate audiological assessment should allow large-scale, multicenter studies in children after COVID-19 infection in order to evaluate the occurrence of CHL and mainly PHI.

The application of protocols for audiological surveillance [43,44] in the COVID-19 era might be recommended not only to better understand HL among the other manifestations of SARS-CoV-2 but for other diagnoses of progressive and late-onset HL otherwise misdiagnosed or diagnosed late in childhood.

## 5. Conclusions

The main clinical features in our sample agree with previous pediatric reports on the presence of mild symptoms in COVID-19 children. HL needs to be considered among the manifestations of COVID-19 in children, nevertheless, we found only cases of transient CHL. However, CHL can affect speech perception and language development in children and needs to be considered for monitoring during and in the post-COVID period. Furthermore, the onset of SNHL during or after COVID-19 infection requires an accurate etiological diagnosis among other causes of PHI through audiological work-ups, including genetic diagnosis, to avoid the risk of misleading other causes of HL. Due to the variety of symptoms and even the long-term consequences of COVID-19, audiological surveillance is crucial to clarify SARS-CoV-2 as a risk factor for PHI.

## Figures and Tables

**Table 1:** Demographic features and characteristics of COVID-19 children.

Parameters	Group A % (n)	Group B % (n)	Group A vs. Group B *p*-Value
Children	27	10	
Gender Male % (n) Female % (n)	48.15 (13) 51.85 (14)	30 (3) 70 (7)	0.46
Age Mean ± SD median (IQR)	months 19.96 ± 8.79 19.0 (14.50, 23.50)	years 11.6 ± 3.86 12.5 (11.25, 13.75)	<0.0001 *
Symptoms % (n)	85.19 (23)	70 (7)	0.36
Symptoms % (n)			
Symptoms % (n)			
Major complications % (n)	7.41 (2)	20 (2)	0.29
Hospitalization % (n)	48.15 (13)	30 (3)	0.46
No treatment % (n)	70.37 (19)	50 (5)	0.27

The table describes the number (n) and the percentage (%) of children in each group (Group A: infants between 0 and <4 years of age; Group B: children between ≥4 and 17 years of age), the gender (M: male; F: female), the age, the number of children with symptoms, complications and hospitalization and the absence of treatment. SD = standard deviation, IQR = interquartile range; * = significant statistical test (*p* < 0.05); Fisher’s exact test was appropriate for comparing all percentages between Group A and B; the Mann–Whitney test was appropriate to compare the median age between Group A and B.

**Table 2:** Treatments of COVID-19 children.

Treatment	Group A % (n)	Group B % (n)
Non-ototoxic antibiotics	7.41 (2)	50 (5)
Paracetamol	11.11 (3)	10 (1)
Steroids	14.81 (4)	40 (4)
Immunosuppressants	0.0 (0)	20 (2)
Heparin	3.7 (1)	40 (4)
Statistical analysis for each group	0.24 (Q)	0.081 (Q)

The table shows the number (n) and the percentage (%) of the most common medicines used to treat patients. The sample is divided into two groups: infants between 0 and <4 years of age (Group A) and children between ≥4 and 17 years of age (Group B). Q = Cochran’s Q test.

**Table 3:** Symptoms of COVID-19 children.

Symptoms	Group A % (n)	Group B % (n)	Group A vs. Group B *p*-Value
Children	27	10	
Fever	70.37 (19)	60 (6)	0.70
Astenia	11.11 (3)	40 (4)	0.07
Vomiting	18.52 (5)	20 (2)	1.0
Diarrhea	18.52 (5)	30 (3)	0.65
Rhinorrea	33.33 (9)	40 (4)	0.72
Sore Throat	14.81 (4)	10 (1)	1.0
Cough	40.74 (11)	30 (3)	0.71
Dyspnea	7.41 (2)	10 (1)	1.0
Rash	7.41 (2)	0.0 (0)	1.0
Hyposmia	0.0 (0)	20 (2)	0.07
Statistical analysis for each group only significant post hoc tests were reported	*p* < 0.001 *(Q) %Fever > % Astenia %Fever > % Vomiting %Fever > % Diarrhea %Fever > % Rhinorrea %Fever > % Sore Throat %Fever > % Dyspnea %Fever > % Rash %Fever > %Hyposmia % Cough > % Hyposmia	*p* = 0.052 (Q)	

The table shows the number (n) and the percentage (%) of the most common symptoms declared among patients. The sample is divided into two groups: infants between 0 and <4 years of age (Group A) and children between ≥4 and 17 years of age (Group B). Fisher’s exact test was appropriate for comparing all percentages between Group A and B; Q = Cochran’s Q test; * = significant statistical test (*p* < 0.05); the Minimum Required Differences method post hoc Q test with Bonferroni *p*-value corrected was used for pairwise comparison of each group. In particular, in the last column, we reported the statistical tests obtained versus comparisons of Groups A and B, while in the last row we reported the statistical analysis for each group.

**Table 4:** Types of hearing loss of COVID-19 children.

Hearing Loss	Group A % (n)	Group B % (n)	Group A vs. Group B *p*-Value (Test)
*SNHL*	0.0 (0)	10 (1)	0.07 (F)
*CHL*	11.11 (3)	30 (3)	
*Absence of HL*	88.89 (24)	60 (6)	

The table reports the number (n) and the percentage (%) of sensorineural hearing loss (SNHL), conductive hearing loss (CHL), and absence of hearing loss (HL) among the two groups in the sample: infants between 0 and <4 years of age (Group A) and children between ≥4 and 17 years of age (Group B). F = Fisher’s exact test.

**Table 5:** Severity of hearing loss of COVID-19 children.

HL Severity	Overall % (n)	CHL % (n)	SNHL % (n)
Mild	57.14 (4)	66.67 (4)	0 (0)
Moderate	42.85 (3)	33.3 (2)	100 (1)
Severe	0 (0)	0 (0)	0 (0)
Profound	0 (0)	0 (0)	0 (0)

The table reports the number (n) and the percentage (%) of the severity of hearing loss (HL) between sensorineural hearing loss (SNHL) and conductive hearing loss (CHL) in the overall sample.

## Data Availability

The data presented in this study are available on request.
